# Triglyceride-Increasing Alleles Associated with Protection against Type-2 Diabetes

**DOI:** 10.1371/journal.pgen.1005204

**Published:** 2015-05-28

**Authors:** Yann C. Klimentidis, Akshay Chougule, Amit Arora, Alexis C. Frazier-Wood, Chiu-Hsieh Hsu

**Affiliations:** 1 Mel and Enid Zuckerman College of Public Health, Division of Epidemiology and Biostatistics, University of Arizona, Tucson, Arizona, United States of America; 2 USDA / ARS Children’s Nutrition Research Center, Baylor College of Medicine, Houston, Texas, United States of America; Georgia Institute of Technology, UNITED STATES

## Abstract

Elevated plasma triglyceride (TG) levels are an established risk factor for type-2 diabetes (T2D). However, recent studies have hinted at the possibility that genetic risk for TG may paradoxically protect against T2D. In this study, we examined the association of genetic risk for TG with incident T2D, and the interaction of baseline TG with TG genetic risk on incident T2D in 13,247 European-Americans (EA) and 3,238 African-Americans (AA) from three prospective cohort studies. A TG genetic risk score (GRS) was calculated based on 31 validated single nucleotide polymorphisms (SNPs). We considered several baseline covariates, including body- mass index (BMI) and lipid traits. Among EA and AA, we find, as expected, that baseline levels of TG are strongly positively associated with incident T2D (p<2 x 10^-10^). However, the TG GRS is negatively associated with T2D (p=0.013), upon adjusting for only race, in the full dataset. Upon additionally adjusting for age, sex, BMI, high-density lipoprotein cholesterol and TG, the TG GRS is significantly and negatively associated with T2D incidence (p=7.0 x 10^-8^), with similar trends among both EA and AA. No single SNP appears to be driving this association. We also find a significant statistical interaction of the TG GRS with TG (p_*interaction*_=3.3 x 10^-4^), whereby the association of TG with incident T2D is strongest among those with low genetic risk for TG. Further research is needed to understand the likely pleiotropic mechanisms underlying these findings, and to clarify the causal relationship between T2D and TG.

## Author Summary

An elevated triglyceride level is generally considered a risk factor for the development of type-2 diabetes. However, recent studies suggest, somewhat paradoxically, that genetic risk for elevated triglycerides may protect against type-2 diabetes. In this study, we test the relationship of triglyceride-associated genetic variants, collectively and individually, with incident type-2 diabetes across three prospective cohort studies comprised of European- and African-American participants. Our findings across studies, racial groups, and statistical models consistently demonstrate that triglyceride-increasing alleles are associated with decreased type-2 diabetes incidence. These genes therefore appear to both increase triglyceride levels and decrease type-2 diabetes risk. More work is needed to understand the physiological mechanism underlying these findings, and to determine the causal relationship between triglycerides and type-2 diabetes.

## Introduction

Along with age, body weight, family history, and other characteristics, triglyceride (TG) level is a major risk factor for cardiovascular disease and type-2 diabetes (T2D). In many prospective studies, TG at baseline is positively associated with T2D incidence, independently of body mass index (BMI) and other risk factors [1], although the direction of causality between TG and T2D is unclear [2], and somewhat ambiguous.

Over thirty single nucleotide polymorphisms (SNPs) have been found to be associated with plasma TG levels through meta-analysis of genome-wide association studies (GWAS) [3,4]. Using a case-control analytical design in over 30,000 individuals, we previously examined the association of 17 genetic risk scores (GRS) for various traits, including lipid traits, with current T2D status (importantly, adjusting only for sex and age), and observed an unexpected, albeit not statistically significant, pattern suggesting that being at high genetic risk for low-density lipoprotein cholesterol, total cholesterol, or TG could be protective against T2D [5]. At the same time, another group found in a cross-sectional study that genetic risk for dyslipidemia is negatively associated with fasting glucose and HbA_1c_ [6] after adjustment for lipid phenotypes. As illustrated and discussed by Li et al. [6], by not adjusting for TG, the negative direct path between TG genes and T2D is weakened by the positive path linking TG genes, TG, and glycemic traits.

Previous studies have shown that *GCKR* variation is associated with both elevated TG and reduced fasting glucose and T2D risk, [7,8] potentially implicating its role in hepatic de-novo lipogenesis [9], through which it would promote glucose uptake, glycolysis, and subsequently increase plasma TG levels [10]. In a Mendelian randomization study, no evidence to suggest a causal role for TG in T2D or related traits was identified, although the authors did identify a “suggestive protective association” of genetically-raised TG levels with T2D risk [11]. Finally, Qi et al. identified a significant positive association between genetic risk for TG and T2D risk. However, in that analysis there was no adjustment for plasma TG levels which would have biased the results towards the null, according to Li et al. [6]. Likely more important, is their exclusion of SNPs with known pleiotropic effects, such as *GCKR* [12].

Here, we seek to further examine these seemingly paradoxical findings by drawing from three prospective cohort studies and across two racial groups, and by considering baseline measures of BMI, high-density lipoprotein cholesterol (HDL-C), low-density lipoprotein cholesterol (LDL-C), TG, fasting glucose (FG) and fasting insulin (FI) as covariates. Specifically, we hypothesize that baseline TG is associated with incident T2D, that genetic risk for TG is negatively associated with T2D incidence, and that the association between TG and incident T2D is stronger among those individuals with low genetic risk for TG.

## Results

Characteristics of the participants in each study are shown in Table 1. In each study, there are more females than males. The number of incident T2D cases ranges from 185 in the Multi-Ethnic Study of Atherosclerosis (MESA) to 789 in the Atherosclerosis Risk in Communities study (ARIC), while the mean follow-up time ranges from 2,844 days in MESA to 9,392 days in the Framingham Heart Study (FHS). Among both EA and AA, we found that the association of the 31-SNP TG GRS with TG was stronger (EA: β = 0.25, p = 7.3 x 10^–204^; AA: β = 0.058, p = 7.8 x 10^–4^) than the association of the 40-SNP TG GRS with TG (EA: β = 0.20, p = 4.8 x 10^–127^; AA: β = 0.042, p = 1.6 x 10^–2^). Therefore, we proceeded with the 31 SNP TG GRS for all subsequent analyses.

**Table 1:** Characteristics of the three samples of European-Americans and two samples of African-Americans.

	European Americans			African Americans	
	ARIC	FHS	MESA	ARIC	MESA
n	7,868	3,430	1,949	2,089	1,149
Age (yr)	54.1 ± 5.7	35.2 ± 9.9	61.9 ± 10.4	53.0 ± 5.7	61.2 ± 10.3
Sex (% female)	54.0	52.7	61.9	62.0	54.7
BMI (kg/m2)	26.7 ± 4.7	25.2 ± 4.2	27.3±5.1	29.2 ± 8.9	29.8 ± 5.9
Plasma triglyceride*	1.46 ± 0.87	300.7 ± 240.8	126.2±71.6	1.2 ± 0.83	98.5 ± 50.3
TG GRS (31 SNP)	154.0 ± 16.4	160.9 ± 16.8	155.4 ± 16.3	160.6 ± 17.5	161.5 ± 17.1
Plasma HDL**	1.34 ± 0.43	51.2 ± 14.7	53.4 ± 16.2.1	1.45 ± 0.45	53.35 ±15.5
Incident cases (n)	789	468	185	383	200
Mean follow-up time (days)	2,859	9,392	2,844	2,529	2,624

*TG units: ARIC—mmol/L; FRAM—Meq/L; MESA—mg/dL

**HDL units: ARIC—mmol/L; FRAM & MESA—mg/dL

### Association of TG, TG GRS, and TG SNPs with T2D incidence

Among EA, TG was strongly positively associated with T2D incidence (hazard ratio (HR) = 1.14, 95% CI [1.09–1.18], p = 8.8 x 10^–11^), conditioning upon age, sex, BMI, and HDL. The association of the TG GRS with incident T2D, including no other covariates, was not statistically significant, but was in the negative direction (HR = 0.997, 95% CI [0.994–1.000], p = 0.06). Upon conditioning for age, sex, and BMI, the TG GRS was significantly negatively associated with T2D incidence (HR = 0.996, 95% CI [0.993–0.999], p = 0.008). Finally, upon conditioning for age, sex, BMI, TG, and HDL, the TG GRS was significantly negatively associated with T2D incidence (HR = 0.991, 95% CI [0.988–0.995], p = 1.2 x 10^–7^). We did not include LDL-C as a covariate as it was not significantly associated with T2D incidence in a model which included BMI, TG and HDL-C. Including LDL-C in the full model resulted in a similar association of TG GRS with T2D (HR = 0.991, 95% CI [0.988–0.994], p = 2.6 x 10^–8^). Including FG and FI as additional covariates resulted in a similar but weaker negative association of the TG GRS with T2D (HR = 0.994, 95% CI [0.990–0.998], p = 3.1 x 10^–3^).

**Fig 1:**
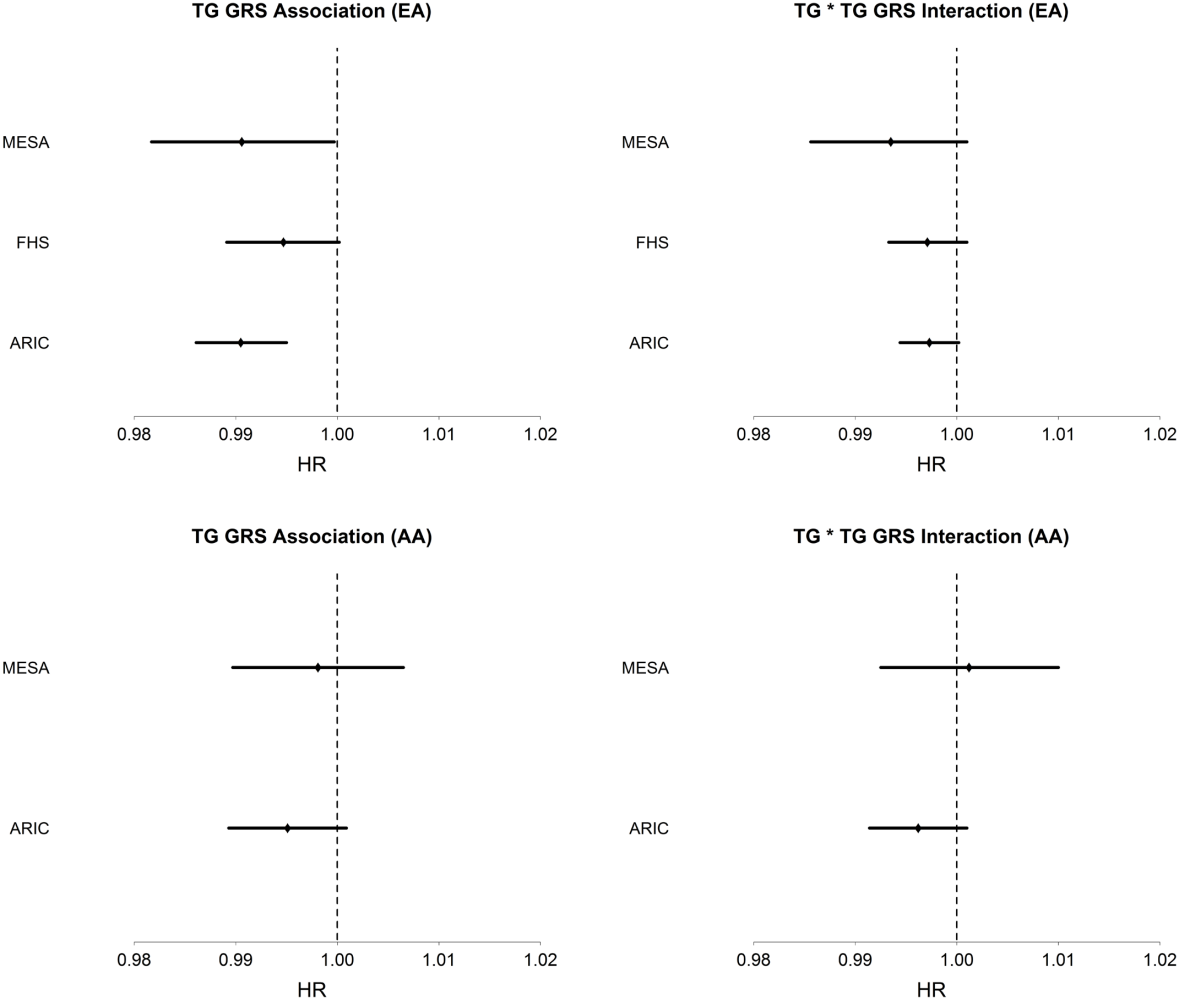
Association of TG GRS with incident T2D and interaction of TG with TG GRS on incident T2D in each dataset and in EA (top; n = 13,247) and AA (bottom; n = 3,238). Hazard ratios and 95% CI are shown. Covariates include age, sex, BMI, TG, and HDL.

Among AA, TG was strongly positively associated with T2D incidence (HR = 1.18, 95% CI [1.12–1.23], p = 3.6 x10^-11^), conditioning on age, sex, BMI, and HDL. The TG GRS was negatively but not significantly associated with T2D incidence (HR = 0.996, 95% CI [0.991–1.001], p = 0.087), conditioning only on age and sex. As in EA, LDL-C was not significantly associated with T2D incidence in any model. Conditioning upon age, sex, BMI, HDL, and TG, the TG GRS association with T2D incidence was negative, but not statistically significant (HR = 0.995, 95% CI [0.991–1.000], p = 0.055). Fig 1 shows the TG GRS association on a study-by-study basis in both EA and AA.

We examined the association of the TG GRS with T2D incidence, conditioning only on race, in a combined dataset of EA and AA. We find that the TG GRS is negatively and significantly associated with T2D incidence (HR = 0.997, 95% CI [0.994–0.999], p = 0.013). Upon conditioning on race as well as age, sex, TG and HDL, we find a very strong negative association of the TG GRS with T2D (HR = 0.993, 95% CI [0.990–0.995], p = 7.0 x 10^–8^).

Of 31 TG-associated SNPs, 24 (77.4%), and 15 (48.4%) are negatively associated with T2D incidence in EA and AA, respectively (see Table 2). However, none are significantly associated with T2D incidence. SNPs that are negatively associated with T2D among EA, at a nominal level of significance (p<0.05) are those in/near *APOA1*, *PLA2G6*, *GCKR*, *CETP*, and *CILP2*. Among AA, no single SNP was at least nominally significant. However, we did observe the same negative trend of association for all SNPs that are nominally significant in EA, except for *PLA2G6*.

**Table 2:** List of 31 triglyceride (TG)-associated SNPs with their respective genomic position, risk allele (TG increasing), TG effect size (weight used in GRS), association with T2D incidence, and interaction with TG phenotype in European- and African-Americans (EA, AA).

				Main Effect on T2D incidence				Interaction with TG phenotype			
				EA		AA		EA		AA	
SNP	Nearest Gene	Risk allele	Effect size	β	p-value	β	p-value	β	p-value	β	p-value
rs10195252	COBLL1	T	2.01	0.1	0.01	0.04	0.44	-0.03	0.23	0.04	0.29
rs10401969	CILP2	T	7.83	-0.16	0.02	-0.09	0.28	-0.06	0.22	-0.06	0.24
rs1042034	APOB	T	5.99	-0.07	0.11	0.07	0.36	-5.8E-3	0.85	0.04	0.37
rs10761731	JMJD1C	A	2.38	-2.7E-3	0.95	0.06	0.33	0.07	0.02	-0.01	0.86
rs11613352	LRP1	C	2.7	0.03	0.46	0.06	0.49	0.04	0.18	0.02	0.79
rs11649653	CTF1	C	2.13	-8.3E-3	0.83	0.07	0.32	-0.01	0.62	0.06	0.08
rs11776767	PINX1	C	2.01	-0.01	0.72	0.05	0.39	-0.02	0.56	0.04	0.35
rs12310367	ZNF664	A	2.42	-0.04	0.33	0.02	0.69	-6.7E-3	0.75	-0.02	0.67
rs1260326	GCKR	T	8.76	-0.08	0.03	-0.04	0.52	-0.02	0.52	0.08	0.07
rs12678919	LPL	A	13.64	-0.03	0.63	-0.11	0.23	-0.04	0.41	-0.14	0.19
rs1321257	GALNT2	G	2.76	-0.01	0.74	-0.04	0.56	-8.7E-4	0.98	0.05	0.21
rs13238203	TYW1B	C	7.91	0.24	0.11	-0.26	0.38	0.07	0.56	-0.2	0.66
rs1495743	NAT2	G	2.97	0.09	0.03	-0.04	0.56	-0.05	0.06	-0.06	0.06
rs1553318	TIMD4	C	2.63	-0.05	0.23	-0.07	0.23	0.03	0.29	-0.06	0.12
rs174546	FADS1-2-3	T	3.82	-0.04	0.3	-0.01	0.88	4.1E-3	0.86	-0.02	0.77
rs2068888	CYP26A1	G	2.28	-3.2E-3	0.94	-0.04	0.57	0.01	0.66	0.12	0.01
rs2131925	ANGPTL3	T	4.94	0.04	0.26	-6.9E-3	0.9	0.03	0.35	-0.05	0.1
rs2412710	CAPN3	A	7	2.1E-3	0.99	0.08	0.59	0.16	0.13	0.02	0.82
rs261342	LIPC	G	2.99	-4.1E-3	0.93	0.04	0.45	-0.06	0.03	0.05	0.19
rs2929282	FRMD5	T	5.13	-0.08	0.33	0.05	0.47	0.21	2.2E-4	-0.04	0.48
rs2943645	IRS1	T	1.89	-0.04	0.27	0.07	0.25	-0.01	0.62	-0.04	0.1
rs2954029	TRIB1	A	5.64	1.7E-3	0.96	6.1E-3	0.92	0.04	0.2	0.02	0.66
rs439401	APOE	C	5.5	-2.8E-3	0.95	0.1	0.18	0.07	0.05	-0.03	0.57
rs442177	KLHL8	T	2.25	-0.02	0.67	0.02	0.79	-0.06	0.08	-0.04	0.34
rs4810479	PLTP	C	3.32	-0.02	0.66	0.03	0.6	-3.1E-3	0.92	-0.06	0.17
rs5756931	PLA2G6	T	1.54	-0.09	0.02	0.13	0.1	-0.05	0.1	0.05	0.24
rs645040	MSL2L1	T	2.22	-9.7E-3	0.83	-0.03	0.6	-0.03	0.43	-0.05	0.31
rs7205804	CETP	G	2.88	-0.1	0.01	-0.07	0.31	-0.03	0.41	0.05	0.27
rs7811265	MLXIPL	A	7.91	-0.05	0.34	-0.09	0.18	0.01	0.72	-0.04	0.14
rs964184	APOA1	G	16.95	-0.15	7.2E-3	-0.08	0.28	-0.06	0.05	-0.04	0.39
rs9686661	MAP3K1	T	2.57	-0.06	0.24	-0.04	0.62	0.07	0.03	-0.07	0.17

Covariates include age, sex, BMI, TG, and HDL.

### Interaction of TG GRS with TG on T2D incidence

**Fig 2:**
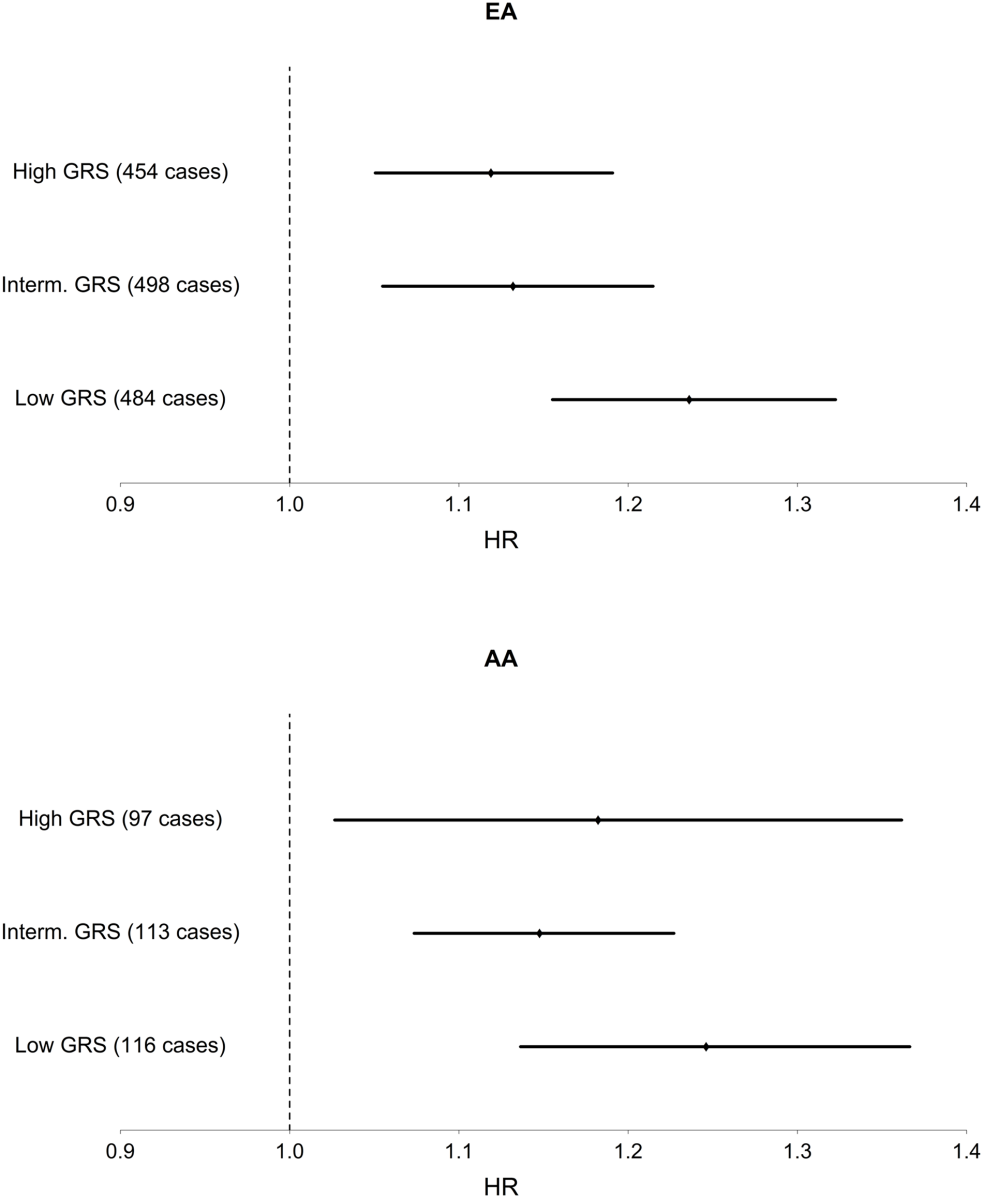
Association of TG with T2D in each of three strata of TG GRS, in EA and AA. Hazard ratios and 95% CI are shown. Covariates include age, sex, BMI, TG, and HDL.

Among EA, we find a significant interaction of TG and TG GRS (HR_*interaction*_ = 0.997, 95% CI [0.995–0.999], p = 1.1 x 10^–3^), with the inclusion of age, sex, BMI, TG and HDL as covariates. Fig 1 shows the TG x TG GRS interaction HRs on a study-by-study basis in EA and AA. Fig 2 shows the association of TG with incident T2D in each of three tertiles of TG GRS. Among EA, we observe a stronger association of TG with incident T2D among individuals with a low TG GRS (HR = 1.24, 95% CI [1.16–1.32], p = 8.0 x 10^–10^) compared to those with a high TG GRS (HR = 1.12, 95% CI [1.05–1.19], p = 4.6 x 10^–4^). Among AA, we do not find a statistically significant interaction of TG and TG GRS (HR_*interaction*_ = 0.998, 95% CI [0.993–1.0003], p = 0.39), although the trend is similar as that in EA (see Fig 2). Including all possible TG x covariate and TG GRS x covariate interaction terms in the model results in a TG x TG GRS interaction that has the same direction as the model without all possible interaction terms, but that is not statistically significant (EA: p = 0.15, AA: p = 0.38, combined: p = 0.06). In a combined analysis, conditioning upon race as well as the other covariates, we find a statistically significant interaction of the TG GRS and TG on T2D (HR_interaction_ = 0.997, 95% CI [0.995–0.999], p = 3.3 x 10^–4^). Among the 31 individual SNPs, only rs2929282 in *FRMD5* shows a significant interaction (p = 2.2 x 10^–4^) with TG on T2D incidence among EA (see Table 2).

## Discussion

Using data from three prospective cohorts, we show that genetic risk for elevated TG is negatively associated with T2D incidence. Furthermore, our findings suggest that the risk conferred by TG is greatest among individuals with low genetic predisposition for TG. We find a similar pattern in both EA and AA, despite 1) the smaller sample size of AA, 2) the fact that TG SNPs were mainly identified in EA, and 3) that the association of the TG GRS with TG among AA in our sample is relatively weak, compared to that in EA.

Our findings are in accord with those of Klimentidis et al. [5] and Li et al. [6]. By adjusting analyses for phenotypic levels of TG and HDL, we were more easily able to identify the negative association of the TG GRS with T2D, and thus confirm the likely pleiotropic nature of TG genes. However, our results differ with respect to the specific SNPs identified. Li et al. identified SNPs in/near *CETP*, *MLXIPL*, *PLTP*, *GCKR*, *APOB*, *APOE-C1-C2*, *CYP7A1* and *TIMD4* as being most strongly negatively associated with glycemic traits. Among these, we find that only *CETP* and *GCKR* SNPs are negatively associated with T2D, albeit at a nominal significance level. All others, except *APOE*, were directionally consistent. Differences in identified SNPs between our study and the Li et al. study may be attributed to, among other things, a difference in the outcome measured (T2D vs glycemic traits). Indeed, the genetic determinants of glycemic traits (i.e. normal glycemic variation) has been shown to differ somewhat from the genetic determinants of T2D [13].

We find that rs964184 near *APOA1* was most strongly negatively associated with T2D, according to the p-value. To our knowledge there is no previous report of an *APOA1* variant being negatively associated with T2D or related trait. The variation near the *APOA1* gene is thought to increase TG levels through impairment of the capacity of APOA1 to bind with lipoprotein lipase (LPL, the enzyme responsible for lipolysis), resulting in decreased LPL-mediated lipolysis of TG [14,15]. In the DIAGRAM meta-analysis, the G allele (TG increasing allele) at this SNP is non-significantly (and positively), associated with a greater risk of T2D (p = 0.15) [16]. We suspect that this non-significant result (and positive direction) is due to the fact that TG is typically not included as a covariate in GWAS of T2D.

Previous studies have shown that variants in *GCKR* may have pleiotropic effects of increasing TG and decreasing insulin resistance [7,8,17–19]. We confirm this in our study. However, our results suggest that *GCKR* is just one of several drivers of the pleiotropic effect of TG genes on T2D risk. A recent functional analysis of *GCKR* variant rs1260326 found that it is highly expressed in hepatic cells in response to glucokinase (GCK) activity, as compared to pancreatic islet cells, indicating its functional role inside liver cells [10,20]. Furthermore, the researchers found the GCK-inhibiting activity, mediated through fructose-6 phosphate, to be significantly attenuated among those with the rs1260326 variant, thus potentially enhancing glucose uptake in the liver and further increasing levels of TG precursor substrate, malonyl-CoA [10]. This influx of glucose inside the liver and resulting increase in the synthesis of malonyl-CoA may account for low glucose and high TG levels. A previous study has also implicated the *CILP2* locus as having opposite putative effects on TG and T2D [21].

The SNP discovery efforts of large GWAS meta-analyses could have down-prioritized SNPs involved in lipid variation among individuals with T2D, if T2D cases were excluded from the analysis. This could potentially have the effect of only discovering SNPs involved in lipid variation among individuals who are non-diabetic. Although T2D cases were excluded from the ARIC study in the Teslovich et al. meta-analysis, for most other studies, T2D cases were included in the analysis, and analyzed separately from controls [3].

These findings have implications for how we understand the causal relationship between TG and T2D. They suggest that additional studies are needed to closely examine the biological and causal links connecting lipid and glycemic phenotypes. For example, several SNPs associated with FI have been found to be associated with higher TG [13,22] providing support for a causal pathway in which a T2D-related phenotype causes elevated TG.

In conclusion, further research is needed to understand the molecular and physiological mechanisms underlying the putative pleiotropic nature of TG-associated genes.

## Methods

### Studies

We used phenotypic and genotypic data of European- and African-Americans (EA, AA) from three prospective cohort studies conducted in the United States for a combined sample size of 13,285. We used data from 7,868 EA and 2,089 AA participants from the Atherosclerosis Risk in Communities (ARIC) study, which is a multi-center prospective study of men and women between the ages of 45 and 64 to investigate risk factors associated with atherosclerosis [23]. We used data from 3,430 EA participants from the Framingham Heart Study Offspring Study (FHS), which is a prospective cohort study to examine the causes of heart disease [24]. Finally, we used data from 1,949 EA and 1,149 AA participants in the Multi-Ethnic Study of Atherosclerosis (MESA), a prospective cohort study of the risk factors for atherosclerosis among middle age men and women aged 45–85 years [25]. This study was approved by the University of Arizona Human Subjects Protection Program (Protocol number: 1300000659R001). No patient consent was given as the data were analyzed anonymously.. Data was obtained from the database of Genotypes and Phenotypes (dbGaP).

### Phenotypic measurements

Individuals with prevalent T2D at the baseline examination were excluded from this study. Prevalent T2D was defined as having a FG level > 125 mg/dL, a report of taking T2D medication, or a physician diagnosis. We also excluded individuals who reported using cholesterol medications, including statins, as these medications can influence blood lipids levels and other blood constituents, which may artificially reduce associations between TG and incident T2D. We also excluded individuals who had not fasted (< 8 hours) at the baseline exam. The samples drawn at the first visit of each study were processed and analyzed using standardized procedures and protocols, the details of which are described elsewhere [26–28]. We used baseline measurements of age, TG, HDL-C, LDL-C, BMI, FG and FI as covariates in the analysis. Height and weight were also measured at baseline, and body mass index (BMI; kg/m^2^) was calculated. Incident T2D cases were identified at one of three follow-up visits in ARIC, seven follow-up visits in FHS, and four follow-up visits in MESA, based on FG, medication criteria, or physician diagnosis. In ARIC, time to incident type 2 diabetes was extrapolated based upon glucose values at the ascertaining visit and the previous visit, as previously described [29].

### Genotypes and genetic risk score

FHS participants were genotyped with the Affymetrix 500K SNP Array (Affymetrix, Santa Clara, CA, USA). ARIC and MESA participants were genotyped with the Affymetrix Genome-Wide Human SNP Array 6.0. Standard quality-control measures were employed prior to imputation. We used IMPUTE2 with the 1,000 Genomes data as a reference, to impute millions of genotypes in each study [30]. To assess genetic risk for elevated TG, we calculated two GRS. The first GRS was based on 31 single nucleotide polymorphisms (SNPs) identified in a large-scale meta-analysis of lipid levels [3]. The second GRS was based on 40 SNPs identified in a more recent meta-analysis of lipid levels [4]. All SNPs in the three studies had imputation quality scores (‘info’) > 0.6. Weighted genetic risk scores were calculated by taking the sum of risk alleles for all SNPs and weighting each risk allele by its respective effect size. Risk alleles and effect sizes were defined according to the findings in the respective meta-analysis.

### Statistical analyses

Since TG, HDL-C, LDL-C, FG and FI levels were measured using different units in each study, we standardized them to a mean of 0 and standard deviation of 1 in each study so that we could combine the studies. Hazard ratios (HR), defined as the ratio of hazard rates corresponding to different levels of TG genetic risk, were estimated using Cox proportional hazards regression models in each of the three studies and in the combined data. We considered the association of TG genes with incident T2D by conditioning on variables including age, sex, BMI, HDL-C, LDL-C, FG, FI, and TG. As mentioned above, phenotypic covariates were measured at the baseline examination in each respective study. Interactions were modeled as the product of TG and the TG GRS (or TG SNP) and included as covariates in the Cox proportional hazards regression model. We also tested a model in which additional interaction terms of both the TG and TG GRS with all other covariates were added to the model, based upon the recommendation of Keller [31]. We combined the studies into one (one for EA and one for AA) to conduct analyses, and accounted for the potential within-study dependence for the subjects from the same study by including a ‘frailty’ term for the study in the model. We also tested proportionality of hazard over time by including a time-dependent covariate consisting of the interaction of the logarithm of the time to event with TG and the TG GRS, and their interaction term. Among EA, there was not sufficient evidence (p>0.05) to reject the null hypothesis of hazard proportionality over time in the TG GRS association or the TG-by-GRS interactions. Among AA, we found the same, except for the TG variable (p = 0.04). However, upon visual inspection and comparison of the Kaplan-Meier survival curves, we observe that the TG >-0.22 (median) and < = -0.22 curves look proportional. We suspect that the p-value<0.05 is attributed to the large sample size. For the SNP analysis, we considered a Bonferroni correction for 31 tests performed, resulting in an alpha of 0.0016. To test the association of each TG GRS with TG, we first natural-log (ln) transformed TG separately in each study, then standardized it as described above. We also standardized each TG GRS in order to compare the association of each with TG. We used linear regression to test the association of each TG GRS with standardized ln(TG) in the set of combined studies, including age, sex, BMI and the random effect of study as covariates. All analyses were conducted with R Statistical Software [32] and SAS Software (Cary, NC).

### Accession numbers

Data for this studies was obtained from dbGaP through accession numbers: phs000007.v23.p8, phs000280.v2.p1, phs000209.v10.p2.
